# Can Post-Exercise Hemodynamic Response Be Influenced by Different Recovery Methods in Paraplegic Sportsmen?

**DOI:** 10.3390/ijerph19031772

**Published:** 2022-02-04

**Authors:** Felipe J. Aidar, Edilson F. Dantas, Paulo F. Almeida-Neto, Frederico R. Neto, Nuno D. Garrido, Breno G. Cabral, Tiago Figueiredo, Victor M. Reis

**Affiliations:** 1 Department of Physical Education, Federal University of Sergipe (UFS), São Cristovão 49100-000, Brazil; fjaidar@gmail.com (F.J.A.); efdantas@hotmail.com (E.F.D.); 2 Department of Physical Education, Health Sciences Center, Federal University of Rio Grande do Norte (UFRN), Natal 59078-970, Brazil; paulo220911@hotmail.com (P.F.A.-N.); brenotcabral@gmail.com (B.G.C.); 3 Paralympic Sports Program, SARAH Rehabilitation Hospital Network, Brasilia 71535-005, Brazil; fredribeironeto@sarah.br; 4 Research Center in Sports Sciences, Health Sciences and Human Development (CIDESD), Trás os Montes and Alto Douro University, 5001-801 Vila Real, Portugal; ndgarrido@gmail.com; 5 Exercise Physiology Laboratory, Estacio de Sá University, Rio de Janeiro 22790-710, Brazil; tiago.figueiredo@ensineme.com.br

**Keywords:** blood pressure, resistance training, cryotherapy, dry needling

## Abstract

Post-exercise hypotension is of great clinical relevance and also in sports training settings, as recovery speed is important. The aim of this study was to compare the influence of different recovery methods on post-exercise hemodynamic response. Twelve male paraplegic sportsmen (25.40 ± 3.30 years) performed a strength training (ST) session using the bench press exercise. After the ST, three recovery methods were randomly performed over a 15-min period: passive recovery (PR), cold-water (CW) and dry needle (DN). Blood pressure (BP), heart rate (HR) and myocardial oxygen were measured before and post ST, as well as post the recovery method. Results: Dry needling induced lower systolic blood pressure (SBP) immediately after the treatment when compared with the other recovery methods, but the contrary was observed at 50 and 60-min post recovery, where records with DN exhibit higher mean values (η^2^p = 0.330). There were no differences in post-exercise diastolic BP and mean BP between recovery methods. There was a significantly higher HR after the PR method, when compared with CW and with DN (η^2^p = 0.426). The same was observed for double product and for myocardial oxygen, though with a larger effect size (η^2^p = 0.446). We conclude that dry needling seems to induce a faster SBP lowering immediately after the procedure but at 50-min post procedure the cold-water method showed better result. As for HR, both procedures (DN and CW) showed a better recovery when compared with passive recovery, along the several moments of measurement.

## 1. Introduction

Bench pressing is the main resistance exercise mode when paraplegic individuals are involved in powerlifting. Bench press is nevertheless one of the most popular strength training exercises and often performed by any person engaged in resistance-exercise programs. The high demand for strength required in powerlifting may incur muscle damage, with a consequent increase in local sensitivity, inflammation, and decreased functionality [1,2,3]. On the other hand, the hemodynamic response may be affected when high-load resistance exercise is performed. Hemodynamics is a set of physiological responses related to the pumping of blood by the cardiovascular system, and includes blood pressure, heart rate, blood flow, among others [4,5].

The balance between recovery periods and stimulation is a paramount requirement for maximizing the effects of continued training. This justifies the growing scientific interest in strategies capable of optimizing the recovery speed between consecutive training sessions [6]. Therefore, trigger point dry needling (DN) and cold-water immersion (CW) are recovery methods used to minimize pain and sensitivity resulting from exercise-induced muscle damage [7].

CW is a technique of immersing the body in cold-water, with temperatures generally below 15 °C. The primary consequence of immersion in cold-water is intense cutaneous vasoconstriction, resulting in decreased peripheral blood flow, which can culminate in a decrease of the body’s central temperature [8].

DN, in turn, is a treatment technique like acupuncture [9]. This technique uses needles to penetrate the skin, subcutaneous tissues, and muscles with the intention of breaking the tissues without using an anesthetic [9,10]. This method promotes increased blood supply around the treated area and can reduce the excitability of the central nervous system. Its effects are associated with decreased spinal nerve activity and attenuation of brain areas associated with pain [10].

Booth these recovery means have been extensively studied. Cold water immersion (CW) has been used to improve pain and injuries, with positive effects on physiological aspects, on the inflammatory process, reducing muscle damage and inflammatory markers, among others. On the other hand, dry needling (DN) has been used to decrease muscle pain, through a local inflammatory process, increasing hyperemia to the injured area. DN has also been used to reduce delayed onset muscle soreness, aiding recovery [6].

Although the physiological mechanisms mediating these effects have not yet been fully clarified, there is evidence that they can cause the breakdown of cardiovascular homeostasis and incur positive effects. Thus, the aim of this study was to compare the influence of different recovery methods on post-exercise hemodynamic response.

## 2. Materials and Methods

### 2.1. Participants and Study Design

Twelve male paraplegic individuals participated in this study and they were recruited among the athletes of a powerlifting team, with mean age of 25.40 ± 3.30 years, body mass of 70.30 ± 12.15 kg and training experience of 2.45 ± 0.21 years. The subjects volunteered to participate in the study and signed the free and informed consent term, according to resolution 466/2012 of the National Research Ethics Commission (CONEP) of the National Health Council and following the ethical principles Declaration of Helsinki (1964, reformulated in 2013), by the World Medical Association. This experiment was approved by the Research Ethics Committee of the Federal University of Sergipe (Technical advice number 2.637.882/May 2018).

All participants were national level athletes; and ranked among the top 10 of their respective categories, according to the criteria of the International Paralympic Committee [4]. As for the types of disability, four athletes had spinal cord injuries due to trauma, with injuries below the eighth thoracic vertebra; two with sequelae of poliomyelitis; four with lower limb malformation (arthrogryposis); and two with cerebral palsy.

**Figure 1:**
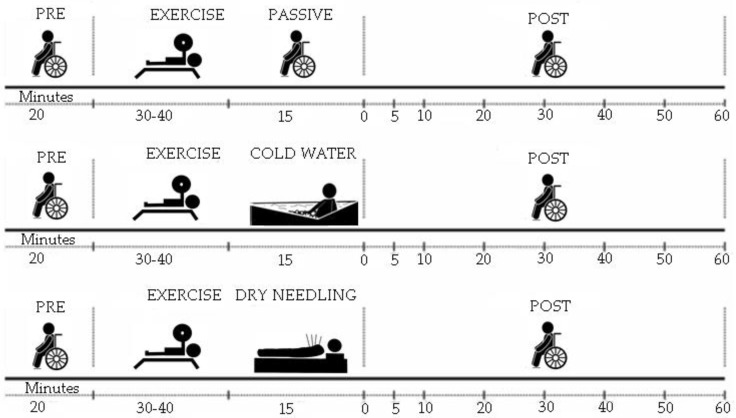
Experimental design.

This study followed a randomized design. Data collection was performed in 4 visits. On the first laboratory visit, the individuals received the guidelines relevant to the study, signed the informed consent form (ICF) and underwent anthropometric assessment and performed the 1 repetition maximum (RM) test. On the subsequent visits, the individuals underwent, in random order, training and evaluation sessions according to the experimental design (Figure 1). The subjects were asked to refrain from the use of nutritional supplements or drugs that could affect the physiological response (For example, Caffeine, alcohol or tobacco).

The training and evaluation sessions were carried out in a randomized order, once a week, always on the same day and time, interspersed with a seven-day interval. The seven-day interval was implemented firstly to assure complete recovery between sessions and then to adjust for the facility and subjects’ availability (no weekend assessments were allowed). Upon arriving at the gym, individuals were welcomed, oriented and directed to the assessment room, where they remained in rest for 20 min to perform the first blood pressure measurement. The temperature of the evaluation room was controlled throughout the evaluation and maintained between 21 and 24 °C. The adapted bench press is the only device intended for Paralympic Powerlifting, where the athlete who lifts the most weight wins [4]. The strength training (ST) session was preceded by a warm-up similar to that performed before the 1 RM test. The ST consisted of 5 sets of 5 repetitions, with 80–90% of 1 RM [4], interspersed with a 5 min rest interval. After ST, individuals were randomly submitted to the passive recovery (PR), cold-water (CW) and dry needling (DN) methods. After these recovery periods of 15 min, the dependent variable measurements were repeated at minute zero and up to the 60 min post-recovery period.

### 2.2. Instruments and Procedures

#### 2.2.1. Anthropometrics and Maximum Force Test

A digital platform scale (Michetti, São Paulo, SP, Brazil) was used. To carry out the Bench Press exercise, an official bank, specific to the sport (Eleiko Sport AB, Halmstad, Sweden), bars and official Olympic rings (Eleiko Sport AB, Halmstad, Sweden) were used.

Before the 1 RM test, the individuals underwent a warm-up consisting of 2 sets of 15 repetitions, with approximately 50% of the maximum suggested load, interspersed with a 2 min interval. The load was increased by approximately 10 and 15% of the estimated total until it could no longer be lifted. Between 3 and 5 attempts were allowed, with a minimum of 5 min interval between each of them [4].

#### 2.2.2. Blood Pressure, Double Product and Myocardial Oxygen

To measure blood pressure, blood pressure monitors Microlife 3AC1-1PC (Microlife, Widnau, Switzerland) were used. Blood pressure (BP) was measured before and after the training session, as described elsewhere [11]. The data on BP are given as systolic blood pressure (SBP), diastolic blood pressure (DBP) and mean blood pressure (MBP). MBP was obtained from the components of SBP and DBP, using the formula $\mathrm{MBP}=\frac{1}{3}\mathrm{SBP}+\frac{2}{3}\mathrm{DBP}$ [11].

The double product amounts the cardiac total work to pump blood through blood vessels and was calculated by HR × SBP [12].

To obtain myocardial oxygen (MVO_2_), we used a mathematical function based on a high correlation between the product of cardiac pressure and MVO_2_. To estimate myocardial oxygen volume (MVO_2_), a mathematical function was used, expressing the result in mL O_2_/100 g ventilations per minute (VE/min), as follows: MVO_2_ = (heart pressure product × 0.0014) − 6.37 [5].

#### 2.2.3. Passive, Cold-Water and Dry Needling Recovery Procedures

Passive recovery was done by having the subjects resting for 15-min in a seated position.

The cold-water procedure was performed in a pool cooled with ice. The water temperature was monitored with a thermometer and maintained at approximately 15 °C. The subjects immersed the body up to the neck for 15 min.

A trained physiotherapist performed the DN technique and explained how it works and how sensations, such as “pinching”, can be perceived during the application due to the stimulus and muscle response. The application site was sterilized using cotton soaked in 70% alcohol, then the individual was placed in the supine position when targeting the pectoralis major (between the third and fourth intercostal space under the midpoint of the clavicle), anterior deltoid (upper, anterior, and 1/3 lateral the clavicle to the deltoid tuberosity of the humerus), and in the lateral decubitus when targeting the brachial triceps (60% distal between the lateral epicondyle of the humerus and the scapular acromial process), where the needle applications were performed by means of stainless and sterile mono-filament (0.25/40 mm), perpendicular to the muscles and held in place for 5 min, not being manipulated or stimulated during the execution of the in situ technique [4].

### 2.3. Statistics

The statistical treatment was performed using SPSS 22.0 (IBM, Armonk, NY, USA). Normality assumption was verified with the Shapiro–Wilk test and followed a normal distribution. Therefore, a two-factor analysis of variance was performed on every dependent variable (SBP, DBP, MBP, HR, DP and MVO_2_) to detect differences between the three-recovery methods × the various moments of assessment. Whenever needed, Bonferroni post hoc was applied. Analysis was considered significant when *p* < 0.05. The partial eta (η^2^p) checked the effect size, where: small effect <0.05, medium effect >0.05 and <0.25, high effect >0.25 and <0.50, and very high effect >0.50 [13,14]. Data are presented as mean ± standard deviations. Based on the effect sizes herein, a post hoc analysis using GPower 3.1.2 (Hinnerup, Denmark) revealed that the amount of 12 subjects in a crossover design enabled a 3 conditions × 9 measurements ANOVA to attain a power of 99%.

## 3. Results

**Figure 2:**
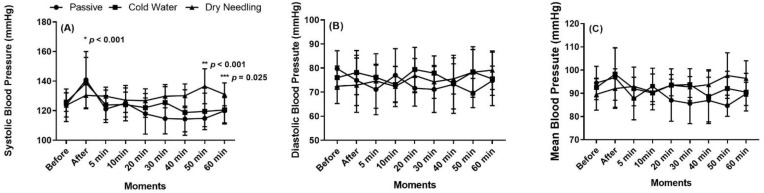
Systolic (**A**), diastolic (**B**) and mean (**C**) blood pressure, before and until 60 min after the training session followed by the three recovery methods. * Difference between dry needling and passive recovery. ** Difference between cold-water and passive recovery. *** Difference between dry needling and passive recovery.

**Figure 3:**
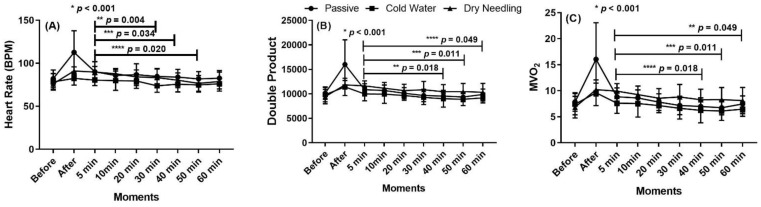
Heart rate (**A**) in Beats for minutes (BPM), double product (**B**) and myocardial oxygen (MVO_2_) (**C**), before and until 60 min after the training session followed by the passive recovery with the three recovery methods. * Difference between passive recovery, dry needling and cold-water. ** Difference with dry needling between minute 5 and minute 40. *** Difference with dry needling between minute 5 and minute 50. **** Difference with dry needling between minute 5 and minute 60.

The results of systolic (SBP), diastolic (DBP) and mean blood pressure (MBP) are shown in Figure 2. The heart rate (HR), double product (DP) and myocardial oxygen (MVO_2_) results are shown in Figure 3.

There was a significant difference in SBP between DN and PR or CW methods, immediately after recovery (η^2^p = 0.330). In addition, there was a significant difference in PR and in CW, at 50 and 60 min after recovery, when compared with DN. Dry needling induced lower SBP immediately after the treatment, but the contrary was observed at 50 and 60 min post recovery.

There was a significant higher HR and double product after the PR method, when compared with CW and with DN (η^2^p = 0.426 and η^2^p = 0.446, respectively, for HR and for DB). In addition, both HR and DB after DN technique significantly lowered at 40, 50 and 60 min, when compared with that at 5 min, with a large effect size. The same differences were detected for myocardial oxygen.

## 4. Discussion

This study evaluated the influence of recovery methods on hemodynamic variables after a strength training session performed by paraplegic sportsmen. The results showed different time-line responses in several hemodynamic variables according to the different post-training recovery methods herein. The main results suggest that both dry needling and cold-water recovery techniques seem promising to speed-up post-training hemodynamics in this subject cohort.

Despite the high intensity of the resistance exercise used in this study (five sets of five repetitions with an intensity of 90% of 1 RM), the hemodynamic response was moderate in every parameter that was assessed, thereby showing a safe cardiac overload herein. In fact, although the load lifted was close to the subjects´ maximum, the low number of repetitions that were performed explains the absence of a heavy cardiac overload [15]. Nevertheless, the recovery of the hemodynamic parameters post-exercise was sensitive enough to separate the three recovery methods that were tested.

To the best of our knowledge, this was the first study to assess recovery of hemodynamics in tetraplegic athletes. As such, the following confrontation with the literature includes those studies that were performed on non-handicap individuals.

The subjects herein were tested in their common movement (bench press), which may help to impair a higher cardiac overload. However, it is possible that the overload could be more prominent if they were required to perform unusual movements. In this case, the recovery methods herein would be of interest due to their potential to speed up the recovery.

### 4.1. Systolic Blood Pressure

During ST, there is a gradual increase in SBP with each repetition that is performed, which tends to increase in subsequent sets, until reaching its peak value at the end of the training session [16]. Typically, after the ST session, the SBP tends to gradually decrease and may reach values transiently lower than those observed before the beginning of the session. This phenomenon is described in the literature as a hypotensive effect of exercise or post-exercise hypotension [17]. A recent meta-analysis reports that different approaches as aerobic exercise vs. resistance exercise (RE) are equally capable of reducing SBP after an exercise session, regardless of the method used or the characteristics of the individuals involved, but that the benefits of exercise on SBP seem to be more effective in hypertensive individuals than in normotensive individuals [18]. Another meta-analysis showed that the post-exercise hypotension magnitude seems to be more related to the amount of muscle mass recruited by the exercise than to the magnitude of the load that is lifted. Different RE intensities were equally able to promote SBP reductions up to 90 min after the end of the exercise session [19], an effect which has persisted for up to 24 h after the exercise [20]. Although this body of knowledge on RE and hypotension, the influence of different recovery methods is not well understood. The results herein showed a significant increase in SBP immediately after the PR and the CW methods were performed (when compared with DN). Indeed, SBP was not shown to be elevated after the application of the DN. However, SBP was still elevated at 50 min and 60 min after application of the DN recovery method when compared with the other methods.

Studies that evaluated the blood pressure response in ST with intensity above 80% of 1 RM, in normotensive men, demonstrated that high intensity is more effective than low intensity to produce post exercise hypotension [21,22,23]. A single study published so far evaluated the blood pressure response of an RE session with an intensity equal to 95% of 1 RM [20]. Its results showed a decrease in SBP for 60 min after the exercise session, which persisted for up to 24 h after the application of the stimulus. Due this long recovery time of hemodynamics post-exercise, we extended the measurements in the current study up-to 1-h post-exercise. In fact, SBP after the various recovery methods at 60 min post-exercise did not follow the same tendency as immediately after each procedure. Especially the DN procedure induced a different late response of SBP, when compared with PR and with CW.

DN is a technique often used to treat muscle pain, and studies investigating its effects on cardiovascular function are still scarce. DN is a western technique based on the insertion of needles in hypersensitive points called trigger points (TP) with the objective of decreasing myofascial pain [24] and is not exactly the same as eastern acupuncture. Acupuncture is a traditional Chinese therapy, which uses the insertion of needles in specific points of the body to restore the balance of forces. Despite the conceptual differences between these two therapies, it is believed that the mechanisms that define the effects of both acupuncture and DN are similar, due to the great similarity between the methods and effects associated with both techniques [9].

Some studies have shown interest in investigating the effects of acupuncture on lowering blood pressure in hypertensive individuals [25]. Still, little is known about the effects on blood pressure of normotensive individuals. The hypotensive effects of acupuncture may be associated with the activation of the nitric oxide syntax in the vascular endothelial cells, promoting an increase in the production of nitric oxide with a consequent decrease in peripheral vascular resistance [26]. In addition to the local vasodilator mechanisms, the central nervous system is also an important mediator of the physiological mechanisms associated with acupuncture [27].

Regarding the effects of CW on SBP, our results differ from a previous study that showed that SBP was significantly higher after DN procedure when compared with passive recovery [28]. In the current study SBP immediately after PR and after CW procedures were both higher when compared with DN. During exercise, there is an increase in blood flow in the active musculature. A greater blood volume is directed to peripheral regions of the body to supply the increased metabolic demand. Immersion in cold-water causes rapid peripheral vasoconstriction, redirecting the blood flow back to the central circulation [29]. To minimize heat loss, the peripheral vessels are compressed intensely, decreasing the subcutaneous blood flow and causing the skin temperature to drop to values close to the water temperature [30]. Skeletal muscles are also affected in this process, the lower the water temperature, the greater the loss of muscle heat. Lower temperatures tend to decrease blood volume in muscle tissue, which contributes to an increase in central blood flow, increasing the venous return and the systolic blood pressure. [31]. Altogether, these mechanisms can help to explain the quick effectiveness of the CW procedure to lower post-exercise SBP.

### 4.2. Diastolic Blood Pressure and Mean Blood Pressure

Our measurements on DBP and MBP did not showed significant differences between pre- and post-training moments, regardless of the post-training recovery method that was used. Meta-analysis papers have reported a significant reduction in DBP after resistance exercise with different intensities, which lasted for up to 24 h. These studies have also shown that the reduction in DBP after RE is greater in hypertensive individuals when compared to normotensive individuals [18,19]. Our findings corroborate previous studies that did not show a significant difference in DBP until 60 min after the resistance exercise, although different intensities were compared during the training sessions [20,21,23]. The occurrence of post-exercise hypotension in terms of DBP, does not seem to be related solely to the exercise intensity. Approaches with similar intensity can promote divergent results with operating times that can vary from 10 min to 24 h [20,22]. Regarding the CW effects on the MBP, our results also differ from other studies that demonstrated that MBP measured after CW was significantly higher in relation to rest [31,32], as the increase in peripheral vascular resistance due to cooling tends to increase the SBP and consequently the MBP [33]. We highlight the fact that this is the first study to investigate these recovery methods in tetraplegic sportsmen, which may help to explain why many differences between our and previous data are evident.

### 4.3. Heart Rate

During exercise, HR typically increases to help supply the metabolic requirements by skeletal muscles, in terms of blood delivery. During strength training with high-intensity (i.e., loads at or above 80% of 1-RM) the shortness of time under tension does not enable HR to attain values that would reflect a high cardiac output [11,20]. Indeed, in our study, the mean peak HR did not exceed 120 bpm. Even so, significant differences were detected among the three recovery methods when post-exercise HR was monitored.

Our results demonstrated that, unlike PR, both CW and DN caused a significant decrease in post-exercise HR with a large effect size. Although both DN and CW have shown similar effects on HR, the mechanisms that promote this phenomenon must have different physiological paths. Regarding the effects of CW on HR, our results corroborate other studies that demonstrated that the HR measured after CW was significantly lower than after PR, a clear effect of a reduction in a perfusion area [28].

### 4.4. Double Product and Myocardial Oxygen

There was a significant higher double product after the PR method, when compared with CW and with DN. In addition, the double product measured after the DN technique was significantly lowered at 40, 50 and 60 min, when compared with that at 5 min, with a large effect size. The same differences were detected for myocardial oxygen. Our results corroborate other studies that demonstrated that the increase in HR and SBP promoted an increase in DP that tends to decrease after the end of physical activity [12,20].

### 4.5. Limitations

Despite the novelty and relevance of the study, we have some limitations: (1) sample size; (2) a short period of trial; (3) the athletes’ diet was not controlled during the study period, as well as (4) the athletes’ sleep time before the assessments. In addition, because the subjects herein were all normotensive, the results cannot be extrapolated to hypertension. Therefore, we suggest the inclusion of extra measurements for an extended period and further studies with more people and with more controlled approaches. Additionally, future studies could explore different combinations of resistance exercise variables (number of sets, relative load, rest interval between sets and combination of different exercises).

## 5. Conclusions

This was the first study to compare passive recovery, dry needling recovery and cold-water recovery methods in tetraplegic sportsmen performing high-intensity resistance exercise.

We conclude that dry needling seems to induce a faster recovery of systolic blood pressure immediately post-exercise, but at 50-min post-exercise, the cold-water method showed a better result. As for heart rate, both procedures showed a better recovery when compared with passive recovery, along the several moments of measurement.

These recovery methods (DN and CW) can be useful complementary strategies to manage exercise-recovery, both between consecutive days of training or within the same training day. In exercise types that contain a risk for an elevated BP (ex. high-load resistance exercise), these strategies seem to be of importance to impair possible cardiac overload.

## Data Availability

The data that support this study can be obtained from the address www.ufs.br/GPEPS, accessed on 21 June 2021.

## References

[1] Keogh J.W.L., Winwood P.W. (2017). The Epidemiology of Injuries Across the Weight-Training Sports. Sports Med..

[2] Joao G.A., Evangelista A.L., Gomes J.H., Charro M.A., Bocalini D., Cardozo D., Seixas da Silva D.A., Simão R., Aylton Figueira J. (2014). Effect of 16 Weeks of Periodized Resistance Training on Strength Gains of Powerlifting Athletes. J. Exerc. Physiol. Online.

[3] Paulsen G., Mikkelsen U.R., Raastad T., Peake J.M. (2012). Leucocytes, Cytokines and Satellite Cells: What Role Do They Play in Muscle Damage and Regeneration Following Eccentric Exercise?. Exerc. Immunol. Rev..

[4] Santos W.Y., Aidar F.J., Matos D.G., Van den Tillaar R., Marçal A.C., Lobo L.F., Marcucci-Barbosa L.S., Machado S.D., Almeida-Neto P.F., Garrido N.D. (2021). Physiological and Biochemical Evaluation of Different Types of Recovery in National Level Paralympic Powerlifting. Int. J. Environ. Res. Public Health.

[5] Paz Â.D., Aidar F.J., de Matos D.G., de Souza R.F., da Silva-Grigoletto M.E., van den Tillaar R., Ramirez-Campillo R., Nakamura F.Y., Costa M.D., Nunes-Silva A. (2020). Comparison of Post-Exercise Hypotension Responses in Paralympic Powerlifting Athletes after Completing Two Bench Press Training Intensities. Medicina.

[6] Schimpchen J., Wagner M., Ferrauti A., Kellmann M., Pfeiffer M., Meyer T. (2017). Can Cold Water Immersion Enhance Recovery in Elite Olympic Weightlifters? An Individualized Perspective. J. Strength Cond. Res..

[7] Bleakley C., McDonough S., Gardner E., Baxter G.D., Hopkins J.T., Davison G.W. (2012). Cold-Water Immersion (Cryotherapy) for Preventing and Treating Muscle Soreness after Exercise. Cochrane Database Syst. Rev..

[8] Mawhinney C., Low D.A., Jones H., Green D.J., Costello J.T., Gregson W. (2017). Cold Water Mediates Greater Reductions in Limb Blood Flow than Whole Body Cryotherapy. Med. Sci. Sports Exerc..

[9] Fan A.Y., Xu J., Li Y.-M. (2017). Evidence and Expert Opinions: Dry Needling versus Acupuncture (II): The American Alliance for Professional Acupuncture Safety (AAPAS) White Paper 2016. Chin. J. Integr. Med..

[10] Fernández-de-Las-Peñas C., Nijs J. (2019). Trigger Point Dry Needling for the Treatment of Myofascial Pain Syndrome: Current Perspectives within a Pain Neuroscience Paradigm. J. Pain Res..

[11] Miyai N., Shiozaki M., Yabu M., Utsumi M., Morioka I., Miyashita K., Arita M. (2013). Increased Mean Arterial Pressure Response to Dynamic Exercise in Normotensive Subjects with Multiple Metabolic Risk Factors. Hypertens. Res..

[12] Neto G.R., Sousa M.S.C., Costa e Silva G.V., Gil A.L.S., Salles B.F., Novaes J.S. (2016). Acute Resistance Exercise with Blood Flow Restriction Effects on Heart Rate, Double Product, Oxygen Saturation and Perceived Exertion. Clin. Physiol. Funct. Imaging.

[13] Lakens D. (2013). Calculating and Reporting Effect Sizes to Facilitate Cumulative Science: A Practical Primer for t-Tests and ANOVAs. Front. Psychol..

[14] Cohen J. (1992). A Power Primer. Psychol. Bull..

[15] Gjovaag T., Hjelmeland A.K., Oygard J.B., Vikne H., Mirtaheri P. (2016). Acute Hemodynamic and Cardiovascular Responses Following Resistance Exercise to Voluntary Exhaustion. Effects of Different Loadings and Exercise Durations. J. Sports Med. Phys. Fitness.

[16] MacDougall J.D., Tuxen D., Sale D.G., Moroz J.R., Sutton J.R. (1985). Arterial blood pressure response to heavy resistance exercise. J. Appl. Physiol..

[17] Kenney M.J., Seals D.R. (1993). Postexercise Hypotension. Key Features, Mechanisms, and Clinical Significance. Hypertension.

[18] Cornelissen V.A., Smart N.A. (2013). Exercise Training for Blood Pressure: A Systematic Review and Meta-analysis. J. Am. Heart Assoc..

[19] Casonatto J., Goessler K.F., Cornelissen V.A., Cardoso J.R., Polito M.D. (2016). The Blood Pressure-Lowering Effect of a Single Bout of Resistance Exercise: A Systematic Review and Meta-Analysis of Randomised Controlled Trials. Eur. J. Prev. Cardiol..

[20] João G.A., Bocalini D.S., Rodriguez D., Charro M.A., Ceschini F., Martins A., Figueira A. (2017). Powerlifting Sessions Promote Significant Post-Exercise Hypotension. Rev. Bras. Med. Esporte.

[21] Duncan M.J., Birch S.L., Oxford S.W. (2014). The Effect of Exercise Intensity on Postresistance Exercise Hypotension in Trained Men. J. Strength Cond. Res..

[22] Boroujerdi S.S., Rahimi R., Noori S.R. (2009). Effect of High- versus Low-Intensity Resistance Training on Post-Exercise Hypotension in Male Athletes: Original Research Article. Int. SportMed J..

[23] Saldanha M.A., Vilaça-Alves J., Neto G.R., Novaes J.D.S., Saavedra F., Reis V.M., Rabelo H.T. (2016). Acute Effect of Resistance Exercise Performed at Different Intensities on the Hemodynamics of Normotensive Men. Motricidade.

[24] Furlan A.D., van Tulder M., Cherkin D., Tsukayama H., Lao L., Koes B., Berman B. (2005). Acupuncture and Dry-Needling for Low Back Pain: An Updated Systematic Review within the Framework of the Cochrane Collaboration. Spine.

[25] Abdi H., Tayefi M., Moallem S.R., Zhao B., Fayaz M., Ardabili H.M., Razavi A.-A., Darbandi M., Darbandi S., Abbasi P. (2017). Abdominal and Auricular Acupuncture Reduces Blood Pressure in Hypertensive Patients. Complement. Ther. Med..

[26] Li J., Sun M., Ye J., Li Y., Jin R., Zheng H., Liang F. (2019). The Mechanism of Acupuncture in Treating Essential Hypertension: A Narrative Review. Int. J. Hypertens..

[27] Longhurst J. (2013). Acupuncture’s Cardiovascular Actions: A Mechanistic Perspective. Med. Acupunct..

[28] Yang Y., Chen S.-C., Yang W.-T., Kuo J.T., Chien K.-Y. (2019). Cold Water Immersion Recovery Strategy Increases Blood Pressure Levels after High-Intensity Intermittent Exercise. J. Sports Med. Phys. Fitness.

[29] Ihsan M., Watson G., Abbiss C.R. (2016). What Are the Physiological Mechanisms for Post-Exercise Cold Water Immersion in the Recovery from Prolonged Endurance and Intermittent Exercise?. Sports Med..

[30] Pandolf K.B., Burr R.E. (2001). Medical Aspects of Harsh Environments.

[31] Choo H.C., Nosaka K., Peiffer J.J., Ihsan M., Yeo C.C., Abbiss C.R. (2018). Peripheral Blood Flow Changes in Response to Postexercise Cold Water Immersion. Clin. Physiol. Funct. Imaging.

[32] Hohenauer E., Costello J.T., Stoop R., Küng U.M., Clarys P., Deliens T., Clijsen R. (2018). Cold-Water or Partial-Body Cryotherapy? Comparison of Physiological Responses and Recovery Following Muscle Damage. Scand. J. Med. Sci. Sports.

[33] Minett G.M., Duffield R., Billaut F., Cannon J., Portus M.R., Marino F.E. (2014). Cold-Water Immersion Decreases Cerebral Oxygenation but Improves Recovery after Intermittent-Sprint Exercise in the Heat. Scand. J. Med. Sci. Sports.

